# Predator efficacy and attraction to herbivore-induced volatiles determine insect pest selection of inferior host plant

**DOI:** 10.1016/j.isci.2023.106077

**Published:** 2023-01-28

**Authors:** Mohammed A. Khallaf, Medhat M. Sadek, Peter Anderson

**Affiliations:** 1 Department of Plant Protection Biology, Swedish University of Agricultural Sciences, Alnarp, Sweden; 2 Department of Zoology and Entomology, Faculty of Science, Assiut University, Assiut, Egypt

**Keywords:** Ecology, Biological sciences, Zoology, Animals, Ethology

## Abstract

Unlike mammals, most invertebrates provide no direct parental care for their progeny, which makes a well-selected oviposition site crucial. However, little is known about the female evaluation of opportunities and threats during host selection. Leveraging the wide range of host plants used by the polyphagous pest, *Spodoptera littoralis*, we investigate oviposition choice between two plants of different nutritional quality. Females prefer to lay their eggs on the host plant, which has inferior larval development and more natural enemies but provides lower predation rates. On the superior host plant, a major predator shows more successful search behavior and is more attracted to herbivore-induced volatiles. Our findings show that predator efficacy and odor-guided attraction, rather than predator abundance, determine enemy free space. We postulate that predators' behaviors contribute to the weak correlation between preference and performance during host plant selection in *S. littoralis* and in polyphagous insects in general.

## Highlights

• An herbivore prefers to oviposit on a plant that provides less favorable development
• A predator is more successful in finding the herbivore on the non-preferred plant
• The predator is more attracted to herbivore-induced odors of the non-preferred plant

## Graphical abstract

**Figure:**
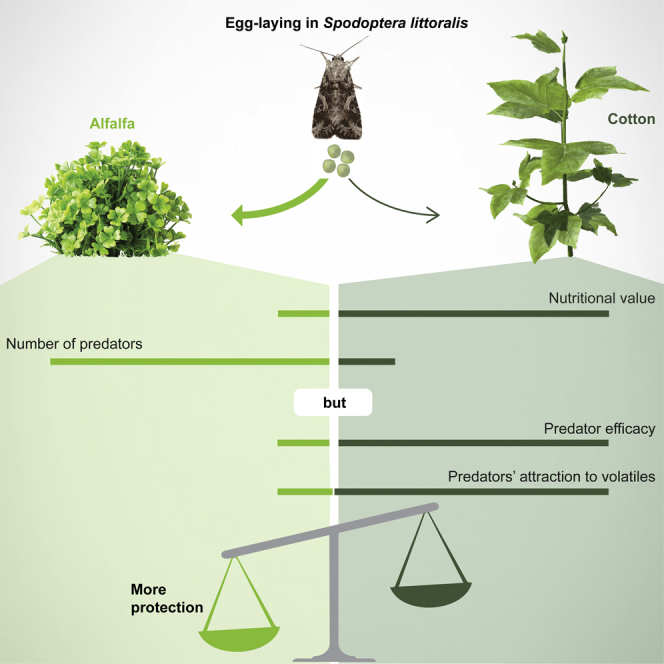

## Introduction

One of the most important and long-standing questions in biology is how parents care for their offspring. Patterns of parental care are widespread and diverse across the animal kingdom; for example, gestation and lactation in mammals,^1^ moisture and heat provision in reptiles,^2^ and active egg guarding against predators in birds.^3^ However, many invertebrates provide no direct care for their offspring, except a limited amount of yolk that serves as an initial food source and a well-selected oviposition site. Furthermore, the ability of immature stages of many invertebrates, to move from one host to another is very limited.^4^ Therefore, the female’s oviposition choice is often crucial for larval growth and survival, and determines the future of the next generation, especially in phytophagous insect species that lay eggs directly on the larval food.^5^^,^^6^ However, despite the numerous documented abilities of phytophagous insects to select optimal egg-laying sites, the mechanisms that underlie such decisions are not well understood.

Phytophagous insect species range from being extreme generalists to being highly specialized in their host use. Generalist phytophagous insects utilize a wide range of plant species and normally show a lower correlation between female oviposition preference and larval performance.^4^^,^^7^ Their oviposition strategy could be seen as a complex trade-off between many, and sometimes contradictory factors. This has been proposed in the “mother knows best principle,” where the female choice of host plant for oviposition is not only governed by offspring performance but is also influenced by other factors, such as their survival on the selected plant.^4^^,^^8^^,^^9^^,^^10^ Furthermore, oviposition choice can also be influenced by factors benefiting survival and performance of the ovipositing female, referred to as the “optimal bad motherhood” principle.^11^^,^^12^ Unlike specialist insects whose wrong choices could be fatal, errors in the process of host plant selection by generalists are less severe due to their larger diet breadth. This makes generalists an appropriate model for investigating how different factors, including natural enemies, shape their behaviors concerning oviposition site selection.^13^^,^^14^

Generalist phytophagous insects may choose a nutritionally poor host plant that provides greater protection for their offspring from natural enemies, known as the “enemy-free space hypothesis.”^10^ In such a case, the fitness reduction resulting from the development of a nutritionally poor host will be balanced by the higher protection provided on the host plant.^10^ Investigations studying the "enemy-free space hypothesis" have mostly concentrated on the influence of parasitism^15^^,^^16^^,^^17^^,^^18^^,^^19^ and pathogens^20^^,^^21^^,^^22^^,^^23^ on host plant selection. However, the role of predators in shaping the host plant choice and oviposition behaviors of phytophagous insects and the mechanism behind it remains less understood.

Multitrophic interactions—including interactions between host plants, herbivorous insects, and their natural enemies—are assumed to largely determine the behavior and the degree of success of both herbivores and their enemies.^24^^,^^25^^,^^26^ Herbivore-induced plant volatiles (HIPVs) are produced by plants after herbivore attack and represent long-distance cues that can provide natural enemies, including predators, with specific information on the presence, identity, and number of prey on a plant.^27^^,^^28^ Recent studies have shown that HIPVs influence the behavior of both herbivores and their natural enemies and result in herbivores’ preference for host plants that provide enemy-free space.^19^^,^^29^ Interestingly, not only herbivore feeding but also the mere deposition of insect eggs on the plant leaves is sufficient to induce the production of oviposition-induced plant volatiles (OIPVs) that attract natural enemies of eggs.^30^^,^^31^ Therefore, HIPVs and OIPVs can be important for predator foraging tactics and a better understanding of how these olfactory cues shape the predators’ decisions.

The Egyptian cotton leaf worm, *Spodoptera littoralis* (Boisduval) (Lepidoptera: Noctuidae), is a generalist moth that represents a good model for studying host plant selection. This species is a serious and widespread polyphagous pest that is found in Africa, Mediterranean Europe, and the Middle East, with a host range including more than 120 species of host plants belonging to 44 families. Females of *S. littoralis* exhibit innate preference hierarchies for egg-laying host selection,^32^ that can be modulated by larval and adult experiences,^33^^,^^34^ and the presence of natural enemies.^7^ One of the known voracious and effective predators of *S. littoralis* eggs and newly hatched larvae is the seven-spotted ladybird beetle, *Coccinella septempunctata*,^35^^,^^36^ which is also known to consume non-prey foods including con- and heterospecifics, and pollen,^37^ Ladybird beetles have been shown to be able to detect and respond behaviorally to HIPVs.^38^

Here, we monitor the diversity of natural predators inhabiting cropping land cultivated with different host plants of *S. littoralis* and investigate the relationship between oviposition preference and offspring performance on these hosts. Moreover, we investigate how HIPVs affect the search behaviors of the predator, *C. septempunctata*. Finally, we link the laboratory work with field investigations to assess the predation risk imposed by arthropod predators on the eggs and pupae of *S. littoralis* and evaluate how far such risk can affect the oviposition behavior of a polyphagous pest.

## Results

### Despite their impaired performance, *S. littoralis* females prefer to oviposit on alfalfa rather than cotton

**Figure 1:**
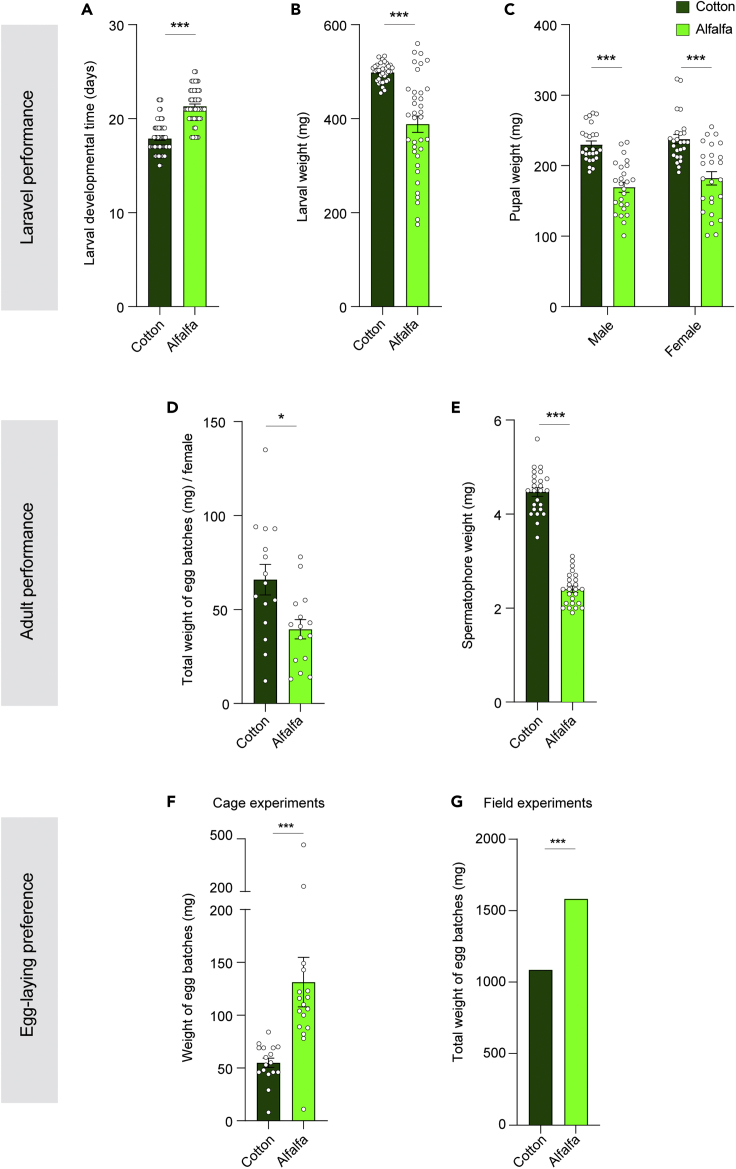
A weak relationship between performance and oviposition preference in *S. littoralis* (A) Effect of host plant on larval development time. Mann-Whitney U test was used (∗∗∗p < 0.001; n = 69 and 58 for larvae reared on cotton and alfalfa, respectively). Unless otherwise noted, in this and other panels, results are given in the form of mean ± standard error of mean. (B) Effect of the host plant on weight increase in the larvae on the fifteenth day after hatching. Unpaired t test was used (∗∗∗p < 0.001; n = 36). (C) Effect of host plant on pupal weight gain. Mann-Whitney U test was used (∗∗∗p < 0.001; n = 25). (D) Effect of host plant on the fecundity of *S. littoralis* in non-choice experiment. Mann-Whitney U test was used (∗p < 0.05; n = 15). (E) Effect of host plant on spermatophore weight. Unpaired t test was used (∗∗∗p < 0.001; n = 25). (F) Two-choice oviposition preference of *S. littoralis* for live intact alfalfa and cotton plants in cage experiments. Wilcoxon matched-pairs signed rank test was used for paired differences (∗∗∗p < 0.001; n = 17). (G) Oviposition preference of *S. littoralis* for alfalfa and cotton in the field. Fisher exact test was used (∗∗∗p < 0.001). Bars represent the total weight of egg batches collected in cotton and alfalfa plots, respectively, over the period of 12 days.

Insect performance—in terms of larval and pupal weight gain, and fecundity—for insects reared on cotton and on alfalfa plants was investigated. Insects raised on cotton had a shorter larval period and heavier weight than those raised on alfalfa (Figures 1A and 1C). Likewise, females grown on cotton deposited a higher total amount of eggs than those raised on alfalfa (in a no-choice assay) (Figure 1D). The spermatophores of the moths raised on cotton were also much heavier than those of the moths raised on alfalfa (Figure 1E).

Next, we assessed the innate oviposition preference of *S. littoralis* in the laboratory for predator-free cotton and alfalfa plants. In addition, in nature, we counted the number of egg batches in cotton and alfalfa plants, which are laid by released females along the borderline between cotton and alfalfa plots (See Material and Methods for details on the experimental protocols). Inconsistent with the better performance of *S. littoralis* offspring on cotton compared to alfalfa (Figures 1A and 1E), lab and field experiments revealed that mated females laid significantly more egg batches on the alfalfa plants than on the cotton plants (Figures 1F and 1G).

### Alfalfa plants harbor more natural enemies than cotton plants

**Figure 2:**
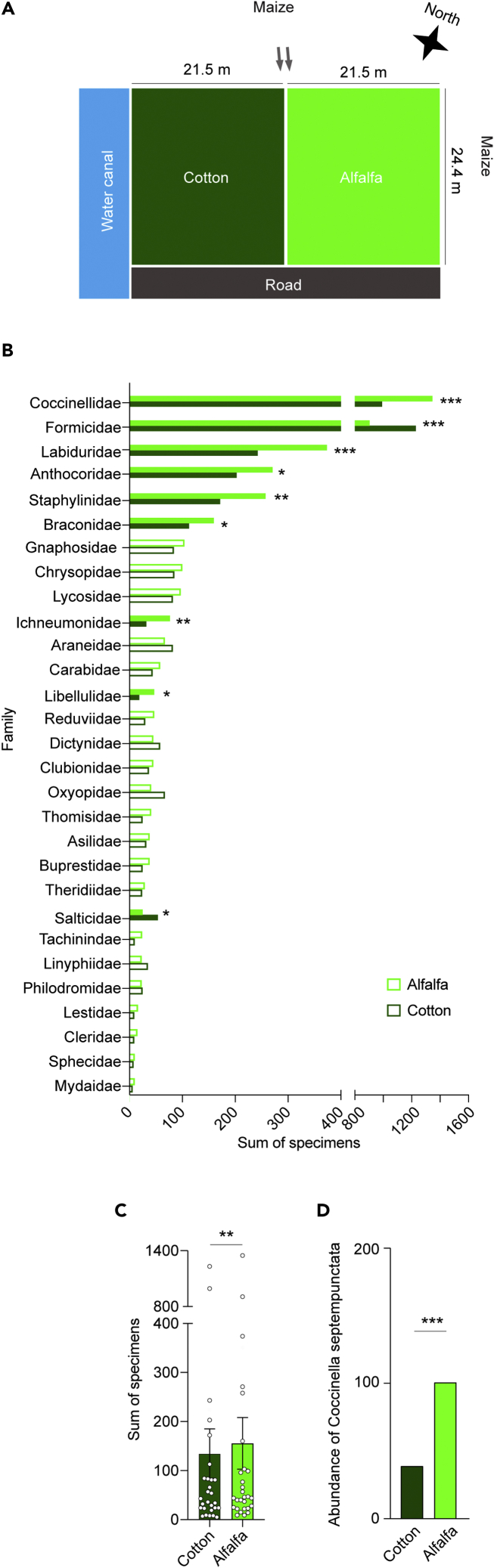
Alfalfa harbor more natural enemies than cotton plants (A) Diagrammatic drawing of the experimental field and surrounding. To reduce the impact of surroundings, the cotton and alfalfa plots were swapped in the following season. (B) The diversity and the number of natural enemies in cotton and alfalfa field. The specimens were collected by sticky traps, pitfall traps, and whole plant inspections in two successive cultivating seasons (2011 and 2012). Families are ordered according to the total number of specimens in each family. Filled bars indicate significant difference between the sum of specimens collected in cotton and alfalfa plots. Fisher exact test was used (ns, p > 0.05; ∗p < 0.05; ∗∗p < 0.01; ∗∗∗p < 0.001). See Table 1 for more details on the number and classification of specimens. (C) The total number of specimens in each family in the cotton and alfalfa field plots. Wilcoxon matched-pairs signed rank test was used for paired differences (∗∗p < 0.01; n = 28 families). (D) The total number of *Coccinella septempunctata* adults in the cotton and alfalfa field plots. Fisher exact test was used (∗∗∗p < 0.001).

An explanation for this weak preference-performance relationship is that *S. littoralis* females may choose a nutritionally poor host plant that provides greater protection for their offspring from natural enemies, known as the “enemy-free space hypothesis.”^10^ We further investigated this hypothesis and whether cotton or alfalfa plants would provide greater protection for the offspring of *S. littoralis* females. Through a combination of different survey methods—sticky traps, whole-plant inspections, and pitfall traps, we monitored the diversity and the number of natural enemies in field plots cultivated by cotton and alfalfa plants (Figure 2A) during two successive cultivating seasons (See Material and Methods for details on the field description). The data from the two seasons were pooled as they produced similar results (Table 1). A total of 8,120 arthropod specimens (particularly insects and spiders), most of which are natural enemies of Lepidoptera, were collected from the cotton and alfalfa plots (Table 1). All specimens were identified at the family level. The specimens belonged to 30 different arthropod families. Six families encompass known parasitoids of Lepidoptera, whereas the remaining 22 families comprise predators that attack many insects, including Lepidoptera. Five families were abundant and comprised more than 200 collected individuals in both cotton and alfalfa plots during both years, namely Coccinellidae, Formicidae, Labiduridae, Anthocoridae, and Staphylinidae (Figure 2B and Table 1). In total, alfalfa harbored significantly more natural enemies than cotton, in terms of the total number of specimens (Figure 2C), which is inconsistent with the enemy-free space.

**Table 1:** The diversity and the number of natural enemies in cotton and alfalfa fields, related to Figure 2

Order	Family	Type	2011		2012	
			Cotton	Alfalfa	Cotton	Alfalfa
Araneae	Araneidae	Predator	39	30	42	36
	Clubionidae	Predator	21	23	15	21
	Dictynidae	Predator	28	20	29	24
	Gnaphosidae	Predator	42	39	41	64
	Linyphiidae	Predator	24	11	10	11
	Lycosidae	Predator	50	44	31	52
	Oxyopidae	Predator	29	16	37	24
	Philodromidae	Predator	11	10	13	12
	Salticidae	Predator	25	12	29	13
	Theridiidae	Predator	14	14	9	14
	Thomisidae	Predator	11	23	13	17
Coleoptera	Carabidae	Predator	19	28	24	29
	Cleridae	Predator	4	8	4	6
	Coccinellidae	Predator	544	763	449	585
	Staphylinidae	Predator	77	135	95	123
Dermaptera	Labiduridae	Predator (omnivorous)	139	200	104	174
Diptera	Asilidae	Predator	21	23	10	14
	Mydaidae	Parasitoid	2	7	3	2
	Tachinindae	Parasitoid	6	16	3	7
Hemiptera	Anthocoridae	Predator	117	137	86	134
	Reduviidae	Predator	16	19	13	27
Hymenoptera	Braconidae	Parasitoid	49	75	64	85
	Formicidae	Parasitoid	629	477	602	429
	Ichneumonidae	Parasitoid	17	40	15	37
	Sphecidae	Parasitoid	4	5	3	4
Neuroptera	Chrysopidae	Predator	34	48	50	51
Odonata	Lestidae	Predator	3	7	5	8
	Libellulidae	Predator	6	19	13	28

Number and classification of specimens collected from cotton and alfalfa fields in the years 2011 and 2012.

### *S. littoralis* females neglect the presence of predators in alfalfa, but not in cotton

**Figure 3:**
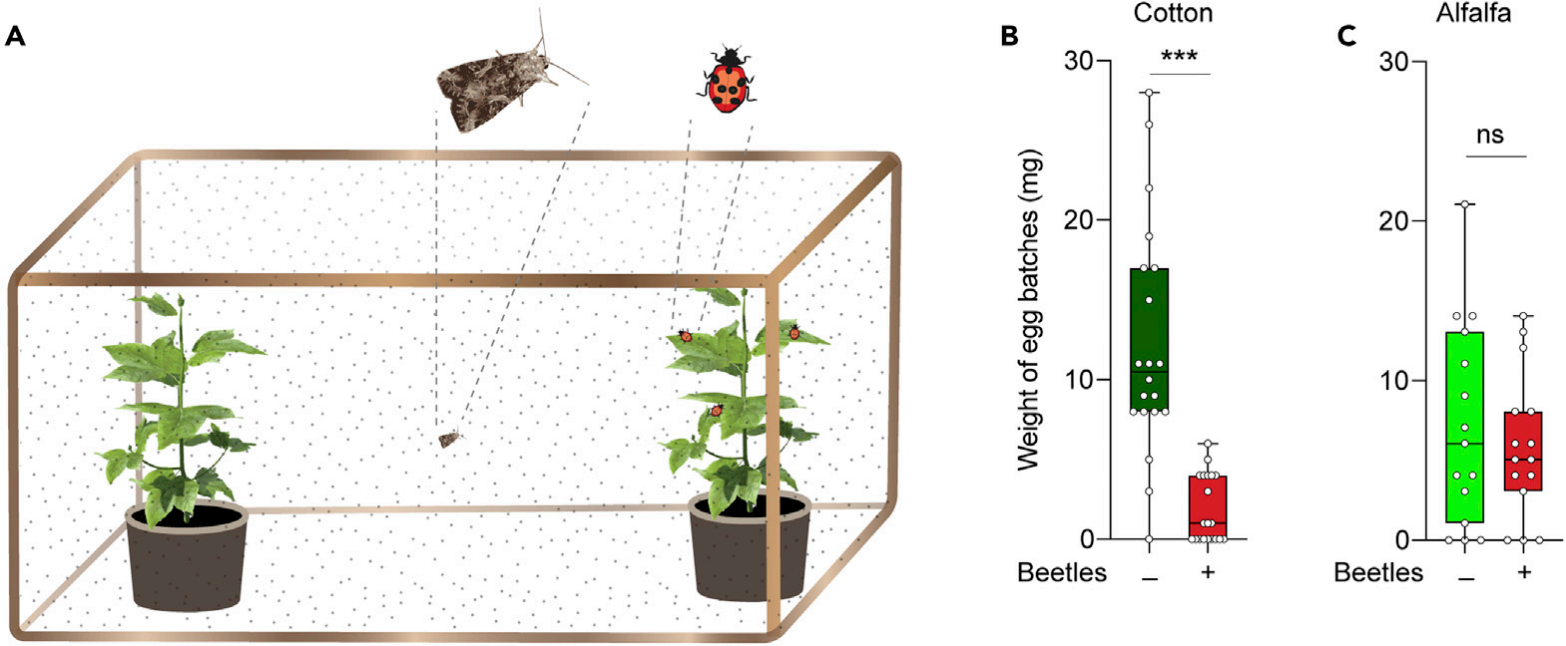
*S. littoralis* females seek predator-free spaces as egg-laying sites in cotton, but not in alfalfa plants (A) Illustration of the setup used to test for the oviposition preference by mated female *S. littoralis* for plants without predators and cotton plants with predators, illustrated by a photograph of a tethered beetle on a plant leaf. (B) Weight of egg batches laid by mated female *S. littoralis* on cotton plants without or with predators. Wilcoxon matched-pairs signed rank test was used for paired differences (∗∗∗p < 0.001; n = 20). (C) Weight of egg batches laid by mated female *S. littoralis* on alfalfa plants without or with predators. Wilcoxon matched-pairs signed rank test was used for paired differences (ns, p > 0.05; n = 15).

We assessed the impact of the predators’ presence on the selection of oviposition sites by giving the gravid females the choice to lay eggs on either plant free from predators or plants that had tethered predators (Figure 3A). The seven-spotted ladybird beetle, *C. septempunctata*, which was more prevalent in the alfalfa field (Figure 2D), was used as a model predator since the Coccinellidae family was the most abundant natural enemy in the field survey (Figure 2B). Gravid females exhibited a highly significant preference for the cotton plants without predators present, compared to the cotton plants with predators existed (Figure 3B). However, gravid females exhibited a non-significant trend to prefer predator-free alfalfa (Figure 3C).

### Predation rates of eggs and pupa are higher in cotton plants

**Figure 4:**
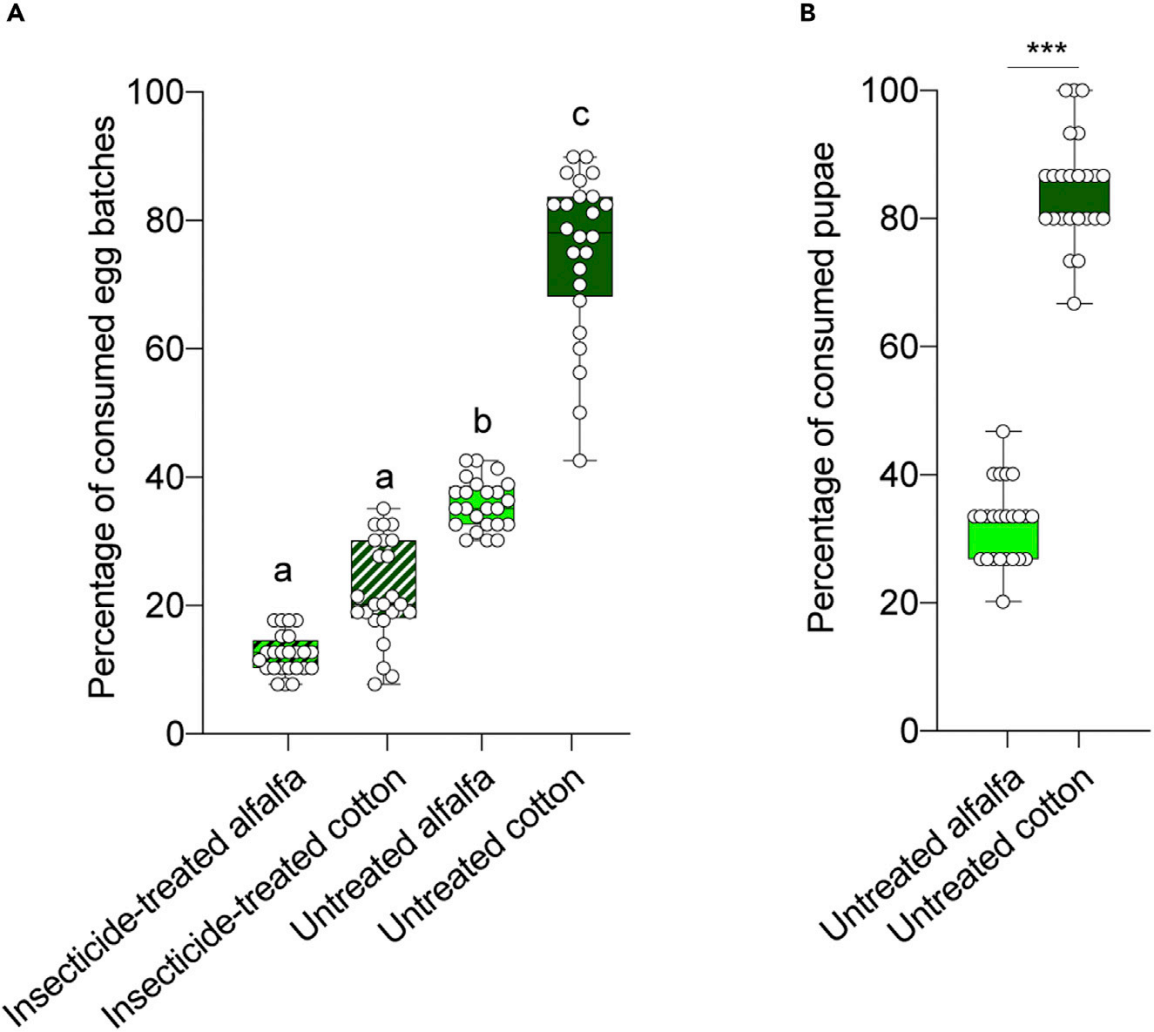
Predation rates of eggs and pupa are higher in cotton plants (A) Egg predation levels in the field. The experiments were conducted three times a month for five months in 2011 and three months in 2012. Letters indicate the significant difference among the treatments calculated by one-way ANOVA followed by Kruskal-Wallis test. Ns, p > 0.05; ∗p < 0.05; ∗∗p < 0.01; ∗∗∗p < 0.001 (n = 24 replicates for each treatment). (B) Pupal predation levels in the field during the cultivating seasons of 2011 and 2012. Experiments were conducted in a similar number to Figure 4A. Mann-Whitney U test was used (∗∗∗p < 0.001; n = 24 replicates for each treatment).

To further investigate why *S. littoralis* females prefer to lay more eggs on host plants that have low nutritional value and harbor a higher number of natural enemies, we assessed the impact of predation on eggs and pupae in the field. We sprayed half of a field area cultivated with cotton and alfalfa with an insecticide to measure the egg and pupal consumption rates in the presence of low populations of natural enemies (See Material and Methods). Though consumption rates were lowered by insecticide treatment, higher rates of egg mass consumption were seen in insecticide-free cotton plots than in insecticide-free alfalfa sites (Figure 4A). Statistical analyses revealed significantly higher predation rates in cotton compared to those in alfalfa plants. Moreover, the mean of consumed egg batches in the different replicates was highest in control cotton, followed by control alfalfa, followed by insecticide-treated cotton, followed by insecticide-treated alfalfa (Figure 4A).

Similarly, we assessed the predation rates of *S. littoralis* pupa in alfalfa and cotton plots. High predation rates were also noted for pupae in both plots, regardless of insecticide treatment (Figure 4B). Taken together, inconsistent with the higher predators’ abundance in alfalfa compared to cotton plots, predation rates of *S. littoralis* eggs and pupa are higher in cotton compared to alfalfa plots.

### Predators (ladybird beetles) exhibit higher searching efficiency of prey on cotton plants

**Figure 5:**
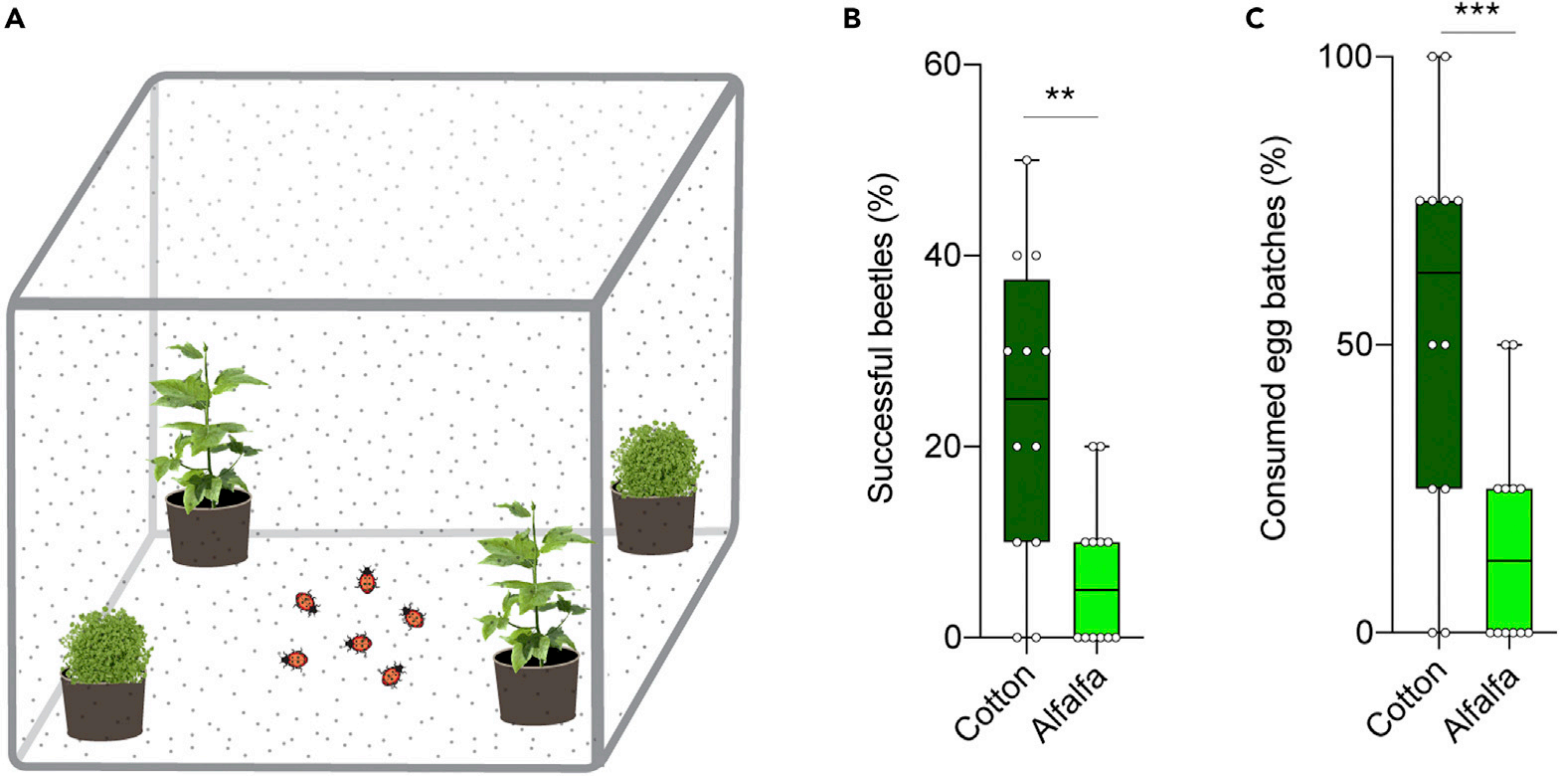
Predators exhibit higher searching efficiency of prey on cotton plants (A) Illustration of the setup used to test how fast predators could find their prey eggs on cotton and on alfalfa. (B) Rate of predator success in finding and consuming the eggs of *S. littoralis* on the two host plants. The proportion of beetles that found the eggs within 6 h from release. Wilcoxon matched-pairs signed rank test was used for paired differences (∗∗p < 0.01; n = 12). (C) The proportion of egg batches consumed by beetles within the 6 h are given. Mann-Whitney U test was used (∗∗∗p < 0.001; n = 12).

Next, we wondered how the higher number of predators resulted in lower predation levels in alfalfa plots and vice versa in cotton plots. Therefore, we examined the efficiency of predators to find *S. littoralis* eggs on the two host plants in the laboratory (Figure 5A). Within 6 h after release into the cages, a significantly higher percentage of beetles succeeded in finding egg batches on cotton as compared to those tested on alfalfa (Figure 5B). By the end of the 6-hour period, more than 50% of the eggs on cotton were consumed, compared to only about 16% of the eggs on alfalfa (Figure 5C).

### Infested cotton plants attract predators more than infested alfalfa plants

**Figure 6:**
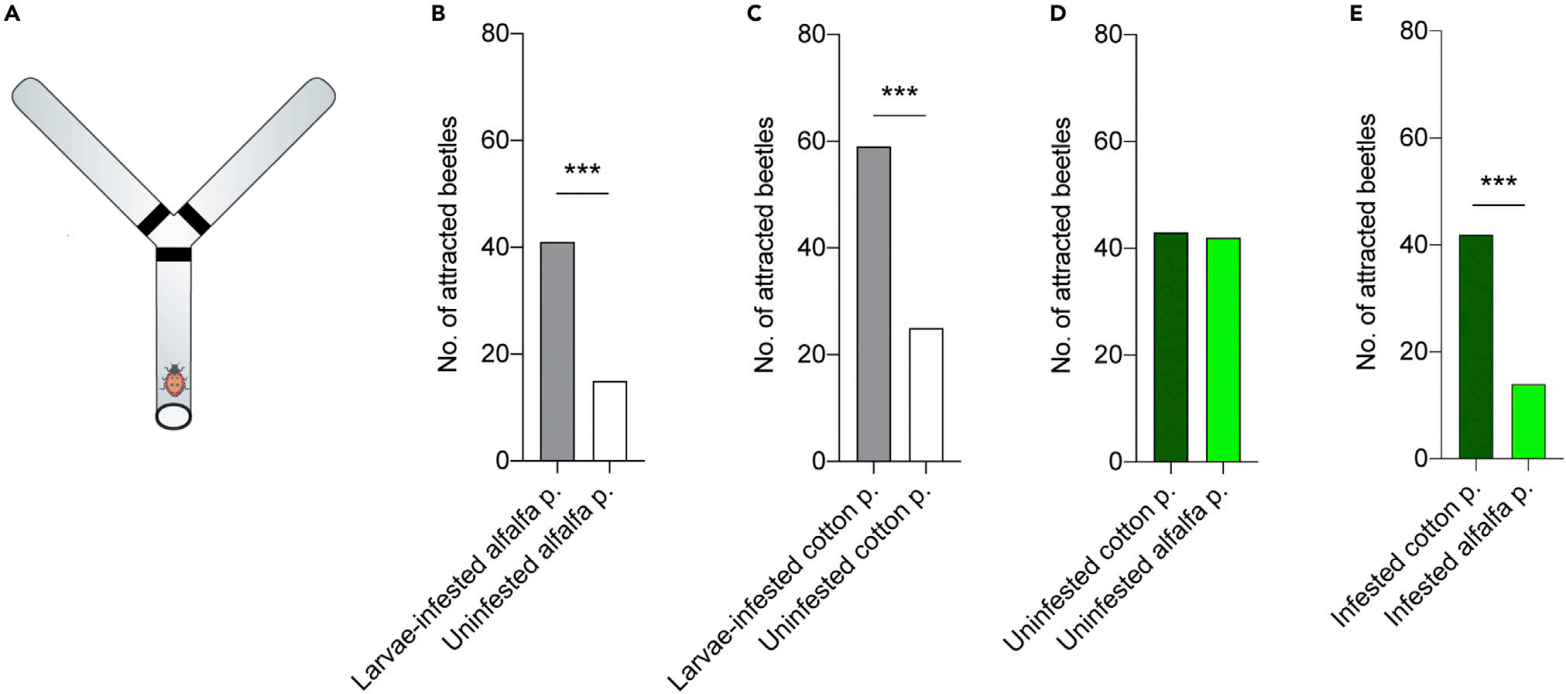
Infested cotton plants attract more predators than infested alfalfa plants (A) Schematic drawing of Y-tube olfactometer and the tested predator beetle. (B) Attraction of predator beetles *C. septempunctata* to larvae-infested and un-infested alfalfa plants. In this and other panels, Fisher exact test was used (∗∗∗p < 0.001; n = 66). (C) Attraction of predator beetles *C. septempunctata* to larvae-infested and un-infested cotton plants (∗∗∗p < 0.001; n = 84). (D) Attraction of predator beetles *C. septempunctata* to un-infested cotton and alfalfa plants. There was no significant difference between the proportion of beetles attracted to cotton and those attracted to alfalfa (ns p > 0.05; n = 85). (E) Attraction of predator beetles *C. septempunctata* to infested cotton and infested alfalfa plants. Preference for cotton was significantly higher than for alfalfa (∗∗∗p < 0.001; n = 56).

Using a short-range attraction assay—a Y-tube olfactometer (Figure 6A)—we examined the olfactory-guided attraction of *C. septempunctata* to odors emitted by different stages of *S. littoralis*, as well as different treatments of plants, and combinations of both. Predatory beetles, *C. septempunctata*, did not exhibit any attraction to *S. littoralis* eggs, larvae, or intact plants (Figure S1A). However, they exhibited strong attraction to larvae-infested plants but not to egg-infested plants (Figures 6B, 6C, S1B, and S1C).

To investigate the impact of the volatiles of cotton and alfalfa plants before and after herbivory on predator attraction, we let predatory beetles choose between un-infested cotton and alfalfa plants. The beetles showed equal attraction to the un-infested plants of the two plant species (Figure 6D). However, when the beetles were given the chance to choose between *S. littoralis-*infested cotton and alfalfa plants, the beetles significantly preferred cotton over alfalfa (Figure 6E). Moreover, removal of the larvae was not sufficient to diminish the elevated attraction toward the infested plants (Figures S1D and S1E), indicating that attraction is attributed to HIPVs and not to larval-emitted odors. Together, predator beetles, *C. septempunctata*, are attracted to herbivore-induced plant volatiles, and they prefer infested cotton over alfalfa cotton plants.

## Discussion

The present study provides evidence of natural enemies as a key factor influencing host plant choice of a generalist herbivorous insect pest in an agricultural habitat. In the field, predation rates of eggs and pupae were significantly higher on cotton, which was superior for larval performance, compared to alfalfa. However, the sum of predators and parasitoids inhabiting alfalfa outnumbered those inhabiting cotton. This indicates a discrepancy between predator abundance and predation levels that could depend on the higher efficacy of natural enemies on cotton. This was exemplified in our experiments on the abundant ladybird beetle, *C. septempunctata,* a predator of *S. littoralis*, that is more efficient in finding herbivore eggs on cotton compared to those on alfalfa. Our experiments also revealed that the predator exhibits higher attraction to herbivore-induced volatiles from cotton than volatiles from alfalfa, showing that olfaction-guided search behavior of the ladybird beetle can be an important factor for the increased efficacy. Our results indicate that *S. littoralis* females choose an inferior host to reduce predation risk and gain higher protection against natural enemies for their offspring. The oviposition preference of female *S. littoralis* is most probably an adaptive behavior resulting in reduced predation and support for the “mother knows best principal.”^4^^,^^8^^,^^9^^,^^10^

In herbivorous insects, attraction to host plants has been shown to be influenced both by the identity and the proportion of volatile plant compounds, including HIPVs, in the blend.^39^^,^^40^ Despite the reduced predator population, cotton volatiles in the field may have been more attractive to the predators of *S. littoralis*, resulting in increased predation rates on cotton. This may depend on quantitative differences in the emission of volatiles involved in beetle attraction. Cotton has been shown to produce larger amounts of herbivore-induced volatiles than alfalfa, making cotton volatiles more readily discernible by natural enemies of herbivores than the volatiles of alfalfa.^41^ Moreover, the difference in predation rates between cotton and alfalfa could be due to qualitative differences in the induced volatiles emitted by the two plant species. For example, in cotton, *de novo* production of induced compounds after insect feeding has been found^42^ and has been proven to affect oviposition choice in *S. littoralis*.^43^

Egg deposition by insects of several orders has been shown to induce responses in plants.^31^^,^^44^ These responses may reduce egg survival, reduce subsequent larval feeding, for example, by altering the expression of defense-related genes, and/or attract natural enemies due to OIPV production.^31^^,^^45^^,^^46^^,^^47^ Our findings, however, indicated that ladybird beetles were not attracted to cotton plants with deposited eggs. Therefore, in our cage experiments, OIPVs may not have influenced predator efficacy to find the egg batches. Instead, when on the plant or in the patch of plants other factors, such as plant architecture, may be more important for prey localization which may also have been the case in our experiments. Nevertheless, it’s plausible that egg deposition can affect subsequent herbivory and/or larval development^48^ and by this the emission of HIPVs and attraction to infested plants.

Plant architecture has been shown to affect the behavior of natural enemies, where simple plant structures often facilitate predator attacks on herbivores.^49^^,^^50^^,^^51^^,^^52^ Consistent with our field observations, alfalfa fields are known to harbor large populations of predators, not only in terms of the number of individuals but also in terms of the number of predator species.^53^ The morphological features of alfalfa plants, with their characteristic dense and stacked leaves, may provide more protected shelters than do cotton plants, whose leaves are flat, open, and less stacked.^54^ This would make it easier for both the juvenile and adult stages of the herbivores to avoid and hide from natural enemies. In our study, direct observation of the behavior of ladybird beetles showed that the predator foraging for egg prey is quicker and more successful on cotton plants than on alfalfa plants. In a similar way, the behavior of more specialist parasitic wasps may also be affected by plant characteristics. In an earlier study, parasitization rates by the specialist parasitic wasp *Chelonus inanitus* on *S. littoralis* eggs were much higher on cotton than on alfalfa.^7^

Increased complexity in the environment, both at the habitat and within-plant level, has been shown to increase the abundance of natural enemies.^55^ As found in our study system, this meta-analysis did not find that the higher abundance of natural enemies was correlated with a higher abundance of prey. Moreover, predators are sometimes subject to predation by other predators and complex environments have been shown to provide better protection against intra-guild predation between different groups of predators.^56^^,^^57^ As for the generalist herbivore *S. littoralis*, safety and survival are also important for its potential predators and influence their distribution and hunting grounds. This could be an explanation for the higher abundance of predators in alfalfa compared to cotton. It is also possible that alfalfa provides a better microclimate and in turn increases the performance and survival of both predators and prey in the field.^55^ Interestingly, however, two families—Formicidae (ants) and Salticidae (jumping spiders)—were exceptionally found more frequently in cotton than in alfalfa, which may be attributed to the specific nutritional requirements and feeding habits of these predators.^58^^,^^59^ As alfalfa does not produce extrafloral nectar, the higher presence of ants in cotton than in alfalfa may be due to the tendency of ants to utilize the extrafloral nectar produced by cotton.

The "enemy-free space” hypothesis predicts that a herbivore insect may choose to lay eggs on a nutritionally poor host plant if this plant provides greater protection from natural enemies than nutritionally richer host plants.^10^ In such cases, the fitness reduction resulting from development on a nutritionally poor host will be balanced by the enhanced protection provided by the host. For example, the mountain apollo butterfly has been found to deposit its eggs 1-2 m away from its preferred host plant as a way of escaping predators.^60^ There are three main conditions that must be fulfilled to characterize an oviposition behavior as aiming toward finding enemy-free space.^61^ First, the fitness of the organism in the presence of enemies must be less than in the absence of enemies. One important factor providing higher fitness is progeny survival. In the present study, we found that the predation of *S. littoralis* eggs was much lower in fields treated with a broad-spectrum pesticide that would have reduced the population of predators considerably in that field. This indicates that predation is a major cause of death in this system. Second, the fitness of an herbivore in the preferred habitat with enemies must be greater than its fitness in the less-preferred habitat with enemies. This term is also fulfilled in the present study, since in the field there was a significantly higher risk of predation on cotton than on the preferred host alfalfa. Third and last, the fitness of the herbivore in the preferred habitat without enemies must be lower than in the less-preferred habitat without enemies. Again, experiments in the present study are consistent with this condition, where the larvae reared on cotton, in the absence of any predators, developed faster and attained larger body weights than those reared on alfalfa, which is the more preferred host plant. Increased female weight has, in many insects, been shown to lead to higher fecundity, which is an important fitness factor.^62^ Thus, the present study provides experimental evidence that the oviposition preference exhibited by *S. littoralis* for alfalfa over cotton is an adaptive behavior aimed at an enemy-free space and is not a haphazard sacrificing of nutritional quality. It also shows that natural enemy efficacy, influenced by induced defense volatiles, differs between potential host plants and can be important when shaping host plant preference.

### Limitations of the study

This study monitored the natural enemies in cotton and alfalfa field. A total of 8,120 arthropod specimens (particularly insects and spiders), most of which are natural enemies of Lepidoptera, were collected from the cotton and alfalfa plots. All specimens were identified at the family level. However, more in-depth identification of the natural enemies could have revealed more information about the role and impact of different predator species.

## STAR★Methods

### Key resources table

**Table:** 

REAGENT or RESOURCE	SOURCE	IDENTIFIER
**Experimental models: Organisms/strains**		

*Spodoptera littoralis*	Assiut University	N/A
*Coccinella septempunctata*	Collected from SLU campus	N/A
*Gossypium hirsutum*	Assiut University	N/A
*Medicago sativa*	Assiut University	N/A

**Software and algorithms**		

GraphPad Prism v.9.2	GraphPad Software Inc.	https://www.graphpad.com
Adobe Illustrator 25.2.1	Adobe Software	https://www.adobe.com

### Resource availability

#### Lead contact

Any requests for resources and information about methodology should be directed to and will be fulfilled by lead contact Mohammed A. Khallaf (mohammed.khallaf@mdc-berlin.de).

#### Materials availability

This study did not generate new unique reagents. Information about the design of the lab and field experiments is available in the method details and upon request to the lead contact.

### Experimental model and subject details

The cotton leaf worm, *Spodoptera littoralis*, was taken from a continuous culture kept in the laboratory on a potato-based diet.^63^ The culture was enriched with field-collected moths at least three times a year. The insects were reared at 25±1°C, 16:8 L:D cycle, and >70% relative humidity. The seven-spotted ladybird adults were collected from boxwood plants (*Buxus sempervirens*) on the SLU campus, Alnarp, Skåne County, Sweden. Males and females were separately kept in 24×18×7 cm plastic boxes at 25°C, 16:8 L:D, and >70% RH. Animals were continuously supplied with 30% honey solution as food.

For laboratory experiments, cotton, *Gossypium hirsutum* and alfalfa, *Medicago sativa*, plants were cultivated in 1.5 L pots in commercial soil (Kronmull, Weibull Trädgård AB, Hammenhög, Sweden) for five to six weeks at 25 ± 2°C and 70 ± 5% RH in a climate-controlled biotron.

For field experiments, cotton and alfalfa were grown in a 1032 m^2^ field (24 × 43 m) near Ma’asara village, about 5 km east of the city of Assiut, Egypt. The area was divided equally between the two plant plots (Figure 2A). In this region wind, if any, is usually very mild all year around. However, to avoid drifting of tested insects, the borderline between the two crops was set parallel to the approximate wind direction, which was determined over a few days using a small flag. The wind direction was also checked during the experiment and was found to be parallel to the borderline between the two plots. The field area was adjacent to a road on two sides, while the other two boundaries were adjacent to maize fields. Before planting, all vegetation was cleared from the field, and the two plants were cultivated according to the standard procedure and irrigation system used by local growers. The field experiments were conducted in two consecutive seasons: 2011 and 2012. It was divided into 4 equal parts: 2 plots of cotton and 2 plots of alfalfa. The cotton plots were adjacent to a water canal, a road, and a maize field, while the alfalfa plots were adjacent to the road, and the maize field (Figure 2A). To reduce the impact of surroundings, the cotton and alfalfa plots were swapped in the following season. Usually, the plants were cultivated in mid-March, and the experiments started on the 3rd of May.

### Method details

#### Insect performance on cotton and on alfalfa plants

Insect performance, in terms of development rate, weight gain, fecundity and spermatophore size, on cotton and on alfalfa was investigated in the laboratory. Neonate larvae were taken from the insect main culture and kept in rearing plastic boxes (5×15×30 cm) with perforated covers. One sample was given alfalfa while the other was given cotton leaves. Both groups were kept under the laboratory conditions mentioned above as rearing conditions. Fresh food was supplied *ad libitum* and the larvae were weighed singly on the 15th day after hatching. At pupation, the larval developmental time was calculated, and the pupae were weighed within 12 h after sclerotization of the pupal cuticle. The pupae were then sexed and left until adult emergence. Two groups of moth pairs (1 male and 1 female) were then kept in 260 mL plastic cups with perforated covers, and were supplied with sugar solution (15%, w/v) on cotton wicks as food. For the first group, the fecundity was recorded. For the 2nd group, the abdomen of each female was dissected immediately after mating to determine the spermatophore weight.

#### The diversity and number of natural enemies in the field

The arthropod natural enemies of Lepidoptera were monitored in cotton and alfalfa plots. Three different methods were simultaneously used to collect these arthropods.^64^ In the first method, eighty pieces (16 × 17 cm) of yellow sticky traps were placed horizontally 1 m above ground and ≥5 m apart from each other in the two plant plots. The traps were collected every 5 days and replaced by new ones. Trapped arthropods were gently released and preserved in 70% ethanol for later identification. In the second method, whole plant inspections were conducted. Five randomly selected cotton plants were removed twice a week by placing a large plastic bag (1.5 × 1 m) over the top of the cotton plant, closing the bottom opening, and cutting the plant at ground level. Bags were brought to the laboratory, where arthropods were collected. In the alfalfa plot, five bundles of plants, each of about the same biomass as a cotton plant, were treated as mentioned for cotton, and were taken to the laboratory to collect the arthropods they carried. In the third method, pitfall traps were used. Twenty plastic jars (20 cm high and 6 cm in diameter) were half-filled with soapy water and were distributed equidistantly in each plot. The jars were inserted deep in the soil such that the jar rim was exactly at the ground level. The traps were replaced with new ones every 3 days. The captured arthropods were extracted from water, washed, and preserved in 70% ethanol for later identification. Due to the large quantity and diverse identities of the species, it was challenging to distinguish the specimens at a level higher than the family rank. The monitoring was carried out from the beginning of May till the end of September in 2011, and from the beginning of July to the end of September in 2012. All identified specimens were adults, and their immature stages might have different feeding habitats.

#### Effect of the presence of predators on oviposition site selection

Oviposition preference tests were conducted inside wire-mesh cages (0.8 m wide, 1.5 m long, and 1 m high) that contained a pair of cotton plants (5–6 leaves). The plants on one side of the cage were kept free from any predators, while the plants on the other side carried predators that were allowed to move freely on the leaves. To prevent the beetles from escaping from one side to the other in the cage, the beetles were tethered to the plants using normal threads. One end of a 10 cm thread was tied around the body of a beetle just behind the coxae of metathoracic legs between the thorax and the abdomen, and the other end was fastened to one plant leaf using sticky tape (Figure 3A). Only one beetle was tethered to each leaf. This procedure allowed the beetles to roam around freely but with no chance to escape. One male and one female pupa were placed in the middle of the cage. The plants were checked twice a day after adult emergence to make sure that both male and female moths had hatched successfully. The number of egg batches was recorded. Cages were visited for at least four successive days.

#### Oviposition preference for cotton and alfalfa plants

Experiments with live plants were conducted in wire mesh cages (1.5 m long, 0.8 m wide, and 1 m high) under greenhouse conditions (25–29°C, 16:8 L:D cycle, and >70% relative humidity) at the Department of Crop Protection Biology at SLU, Sweden. Two cotton plants and two pots, each with several alfalfa plants, were placed on opposite sides of each cage. Five male and five female pharate adults were placed in a Petri dish in the center of each cage and left until adult emergence. After adult emergence, the plants were checked daily for eggs. The number of found egg batches was daily recorded and removed. The cages were visited for five days, starting from the day when the first egg batch was found.

The oviposition preference of *S. littoralis* in the field was examined according to the method described in.^7^ Briefly, 8 groups of pupae (each consisting of 15 males and 15 females) were taken to the field where cotton and alfalfa were cultivated. The groups of pupae were placed at 8 equidistant points along the borderline between cotton and alfalfa plots. The pupae were gently buried about 1 cm deep in the soil and were covered with tight wire-mesh boxes to keep them from predation. Once the first adults have appeared, the mesh boxes are removed for a while before sunset to free the moths and then brought back again to their position. This was done until no adults emerged anymore. Counting the emerging adults every day and retrieving the exuviae later showed that at least 94 females and 89 males had emerged successfully. Before the expected time of adult emergence, the two plant plots were carefully inspected, and any found egg batches were manually picked up and destroyed. This was done to ensure that the greatest majority of eggs to be found on the plants during the experiments were oviposited by the laboratory-reared moths. The transfer of pupae to the field was done on the 10^th^ of May 2009. At that time, the plants were about 7 weeks old. From May 13th on, the plants were checked every two days for egg batches. The procedure lasted for 14 days, during which the egg batches were removed daily and taken to the laboratory for weighing and calculating the number of eggs per batch. The experiment was repeated in 2010 (starting on the 16^th^ of May) with 7 groups of 13 male and 13 female pupae. Out of the 91 pupae, at least 59 male moths and 67 female moths had successfully emerged. The collecting of egg batches lasted for 12 continuous days.

#### The risk of egg and pupal predation in the field

The risk of egg predation in the field was assessed according to the method described in detail by.^65^ The method depends on dividing the field into two plots, after which one plot is treated with pesticides to kill potential natural enemies, and the other is left untreated to keep natural enemies alive. The prey samples are then distributed in the two plots, and the losses in the two plots are compared after a certain amount of time. Following the same method, the field area cultivated with cotton and alfalfa was divided into 4 equal parts, 2 plots of cotton and 2 plots of alfalfa. One plot of each crop was left untreated, while the other was treated with the pesticide, pyrethroid lambdacyhalothrin (Kima® Egypt), and was applied using a backpack sprayer at a rate of 15 g/acre.

Egg batches of *S. littoralis*, oviposited in the laboratory on wax paper, were prepared for the field tests. The pieces of wax paper carrying egg masses of roughly the same size were trimmed to 2 × 2 cm strips, each carrying one egg mass. The strips were taken to the field mostly on the same day of oviposition. In only few cases, the timing of the experiment required storage of eggs and, in these cases, the eggs were stored at 5°C for no more than 5 days^66^ A group of 480 egg masses were distributed among the four plots of the field, 120 egg batches per plot. Wax paper strips carrying the egg masses were fixed to the underside of a cotton or alfalfa leaf, using a metal clip just below the upper third leaf of the cotton plant or roughly the corresponding position on the alfalfa plant. The masses in each plot were distributed in such a way that they had nearly equal horizontal distances between them. Short wooden sticks were inserted in the ground beside each batch-carrying plant to serve as a landmark for facilitating the tracking of batches. Since the effect of pesticide fades away after about one week of application,^67^ spraying the pesticide repeatedly was timed to ensure the maximum effect on the days when egg batches were exposed in the field. In all cases, the application of pesticide was done one day before taking the batches to the field. The egg-carrying strips were attached to the plants right before sunset. Starting from the next day, the egg-carrying strips were daily checked, and the egg losses were recorded.

To make sure that any loss in the eggs was not due to hatching and escaping of larvae, additional egg batches were also taken to the field but held inaccessible to predators, by enclosing them within transparent plastic bags whose openings were twisted and closed around the petioles of batch-carrying leaves. In each plot, five such batches were distributed. These batches were used as sentinel samples that helped to know when, and if, the eggs had hatched. In general, none of the sentinel egg batches remained unhatched longer than 3 days. Therefore, any experimental egg batch that was not found after three days of being in the field was excluded from calculations. The experiment was repeated 15 times during the period from May to September 2011 (3 times per month). A similar set of experiments, with the same number of exposed and sentinel egg masses, was carried out again 9 times in the period lasting from July to September 2012.

Predation on *S. littoralis* pupa was also quantified following the same procedure described by.^65^ However, preliminary tests showed that insecticide application doesn’t affect the level of predation on pupa. Therefore, the cotton plot was treated as one unit, and so was the plot of alfalfa, regardless of where the pesticide was applied. Sixteen groups of late 6^th^ instar larvae, each consisting of 10 individuals, were taken from the laboratory and placed at 8 different locations in each of the 2 field plots. The larvae were left on the ground together with some fresh insecticide-free leaves, covered with perforated plastic cages (6×18×20 cm), and left until they pupated in the soil. After pupation, six of the eight plastic boxes were removed, and the remaining two were left in place to protect the pupae from predation. These covered groups of pupae were used as sentinel samples, giving information on the time of emergence.^65^ Short wooden sticks were also used to mark the sites of pupae. Six days after the putative day of pupation, the soil was inspected carefully and any remaining pupae were collected, where the loss in pupae was calculated. In all cases, information derived from the sentinel boxes indicated that the pupae were inspected 2 days before adult emergence could have happened. Similar to the experiment on eggs, the test was carried out in the two cultivating seasons, 2011 and 2012, at the same time schedule and number of replicates used for egg masses.

#### The readiness of predators to find and consume eggs

Under greenhouse conditions, the ladybird beetle *C. septempunctata* was allowed to choose between eggs of *S. littoralis* laid on cotton and alfalfa inside wire-mesh cages (0.8 m wide, 1.5 m long, and 1 m high). A pair of cotton plants and two pots with alfalfa plants of comparable fresh weights were placed on opposite sides of the cage (Figure 5A). Each cotton plant and alfalfa pot were prepared to carry two freshly oviposited egg batches of *S. littoralis* on two different leaves. Ten adult beetles were released in the middle of the cage after being starved for 12 h, and their behavior was directly observed. To facilitate the observations, only two cages were observed at a time. This was repeated 6 times, making a total sample size of 12 cages. Observation was performed by checking the cage at 10-to 15-min intervals to record the number of beetles that succeeded in finding the batches on either plant and started to eat. The observations lasted for 6 h unless the beetles had already found and consumed the eggs earlier.

#### The olfactory-guided attraction of the predator ladybird beetles

The walking response of adult ladybird beetles, *C. septempunctata*, in response to potential volatiles emitted by different stages of *S. littoralis*, as well as by the plants in various settings, or combinations of both, was examined using a Y-shaped olfactometer according to.^68^ In brief, the olfactometer had two side arms that measured 21 cm in length. The central arm was 23 cm long and the inner diameter of all the arms was 1.5 cm. The angle between the two side arms was 95°. During the test, the olfactometer was fixed on a piece of brown cardboard (44 × 44 cm), with the ends of the two side arms at one horizontal plane that was slightly higher (about 10°) than the level of the beginning of the main arm (Figure 6A). To avoid visual distraction during the experiment, the Y-tube was placed inside a box of white fabric (45 × 45 × 50 cm), and the whole set-up was mounted on a trolley whose position and orientation were adjustable to ensure symmetric light from behind. The first 2 cm of the arms starting from the Y-junction were surrounded with black ribbons to darken the central area where the three arms meet. This is the area where an insect makes a choice. Darkening this area of the Y-tube makes the insects slow down, or even stop for a while, before choosing either arm, thereby decreasing the number of random choices.^68^

The filtered air entered each plastic bag at the bottom and left the bag from the top to reach the arms of the olfactometer (Figure 6A). Ladybird beetles were released individually into the central arm of the olfactometer and allowed to choose. The choice of either arm was recorded, with beetles that walked 8 cm up into the side arm considered to have made a choice. Beetles not making any choice within 10 min were counted as non-responding and were excluded from calculations. To avoid positional effects, the side arm positions were reversed every 5 observations. After each experimental assay, the Y-tube was carefully cleaned with ethanol, followed by heating the tube to 350°C for 30 min in order to remove any contaminating odors. The infested cotton and infested alfalfa plants were prepared by allowing one 3^rd^ to 4^th^ instar larvae of *S. littoralis* to feed on each plant. After two days, one plant of each specimen with larvae was enclosed in a plastic bag in the Y-olfactometer setup and was promptly used for the test. The number of beetles attracted to each plant was recorded.

### Quantification and statistical analysis

All statistical analyses and preliminary graphs were made using the software GraphPad Prism v.9.2. Figures were then processed with Adobe Illustrator 25.2.1. Datasets were checked for normal distribution using the Shapiro-Wilk test at a significance level of 0.05. Unpaired t tests or one-way ANOVA followed by the Kruskal Wallis test were used for comparisons between two or more normally distributed datasets, respectively. Comparisons between two nonparametric groups were done using Mann Whitney U test. Wilcoxon matched-pairs signed rank test was used for paired differences between two nonparametric groups. Fisher’s exact test was used to compare between the different proportions in the oviposition and y-tube olfactometer choice experiments, and between the abundance of the different insect families in cotton and alfalfa fields. The significance level was set at 0.05 and is denoted by asterisks (∗p < 0.05, ∗∗p < 0.01, ∗∗∗p < 0.001).

## Data Availability

• All raw data reported in this paper will be shared by the lead contact upon request. • This paper does not report original code. • Any additional information required to reanalyze the data reported in this paper is available from the lead contact upon request.
