# Supplementary material for: Predator efficacy and attraction to herbivore-induced volatiles determine insect pest selection of inferior host plant
Source: iScience. 2023 Jan 28;26(2):106077. doi: 10.1016/j.isci.2023.106077 (PMC9929603; doi:10.1016/j.isci.2023.106077)
Supplement: Document S1. Figure S1 [file mmc1.pdf]

iScience, Volume *26*

Supplemental information

Predator efficacy and attraction to herbivore-induced volatiles determine insect pest selection of inferior host plant

Mohammed A. Khallaf, Medhat M. Sadek, and Peter Anderson

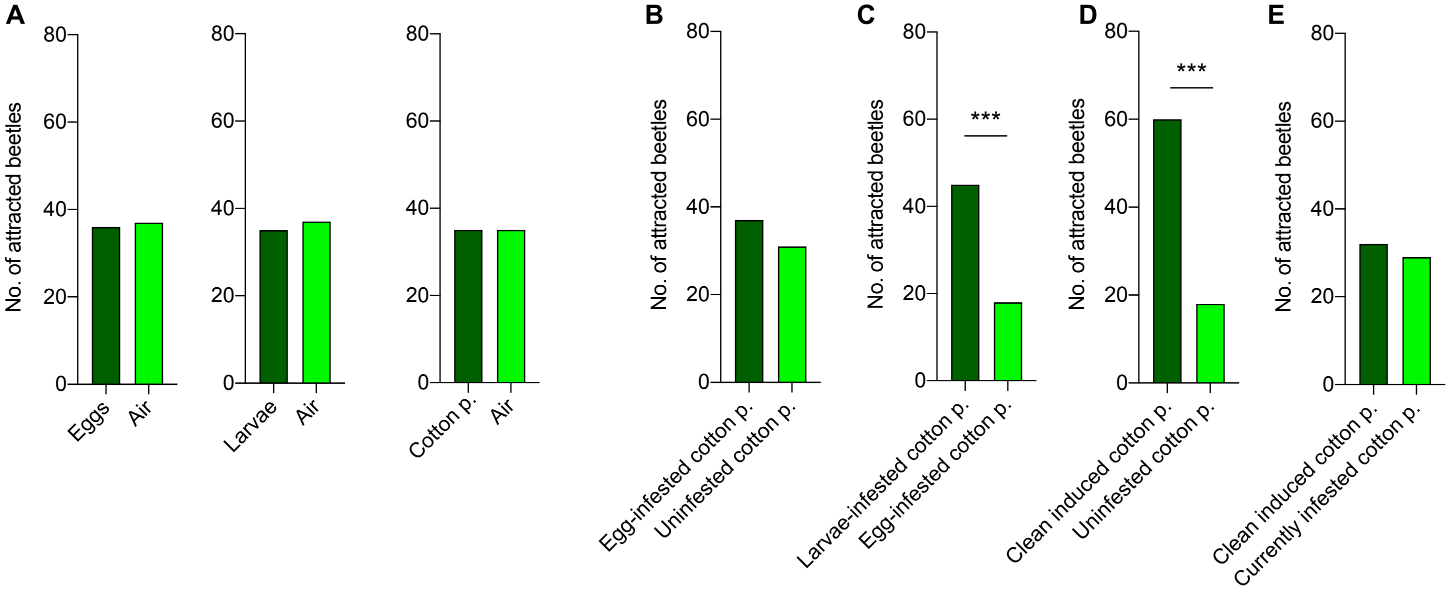

Figure Supplementary 1 (related to Figure 6). Infested cotton plants attract more predators than infested alfalfa plants

(A) Attraction of predator beetles *C. septempunctata* to eggs or larvae of their prey herbivore, *S. littoralis*, or cotton plants and blank air in the Y-shaped olfactometer. In this and other panels, Fisher's exact test was used (ns, $P > 0.05$; n=73, 72, and 70, respectively).

(B) Attraction of predator beetles *C. septempunctata* to egg-infested and un-infested cotton plants (ns, $P > 0.05$; n=68).

(C) Attraction of predator beetles *C. septempunctata* to egg-infested and larvae-infested cotton plants (***$P < 0.001$; n=63).

(D) Attraction of predator beetles *C. septempunctata* to clean induced cotton and un-infested cotton (***$P < 0.001$; n=78).

(E) Attraction of predator beetles *C. septempunctata* to clean induced cotton and currently infested cotton (ns, $P > 0.05$; n=61).
